# Normative study of vocal acoustic parameters from children from 4 to 12 years of age without vocal symptoms. A pilot study

**DOI:** 10.1590/S1808-86942010000400013

**Published:** 2015-10-19

**Authors:** Elaine Lara Mendes Tavares, Roberto Badra de Labio, Regina Helena Garcia Martins

**Affiliations:** a Speech and hearing therapist. PhD student in the Graduate Program of General Basis of Surgery - Unesp-Botucatu; b Speech and hearing therapist and MSc student in the Graduate Program of General Basis of Surgery - Unesp-Botucatu; c Associate Professor - Medical School of Botucatu - São Paulo State University (UNESP). Head of the voice and phoniatry ward. Professor of Otorhinolaryngology at the São Paulo State University, Botucatu Campus

**Keywords:** child, voice disorders, voice

## Abstract

Acoustic vocal analysis is a simple and fast method that allows to differentiate normal from changed voices. There are few studies that analyze normal acoustic vocal parameters at different age ranges in children.

**Aims:**

To establish normative acoustic parameters of children's voice aged 4 to 12 years.

**Methods:**

Two hundred and forty children were divided into four sub-groups by age: G1 (n-60; 4-5 years), G2 (n-60; 6-7 years), G3 (n-60; 8-9 years) and G4 (n-60; 10-12 years). The children's parents answered a questionnaire and the children were submitted to auditory acuity evaluation (Assessment of Transient Otoacoustic Emissions), acoustic vocal analyses, otorhinolaryngological and videolaryngoscopy exams.

**Results:**

The normal values for the acoustic vocal parameters studied were established according to age range and gender. As age increased, there was a decrease of f0 and APQ and an increased in SPI with statistical difference of these parameters. The vocal parameters did not differ between genders until the age of 12.

**Conclusions:**

the characterization of the normative vocal patterns of children is an important reference for future studies. Some of the changes showed a direct relationship between age and a reduction of f0 and of APQ, and increase in SPI, with no difference between genders.

## INTRODUCTION

Since speech is the most important means of communication and expression, any voice disorder can bring about deep implications to social life, both in children and in adults. According to Freitas et al.^1^, a good vocal quality is important for social relations to happen effectively. Della Via^2^ states that voice change during childhood may affect the child's school, social and emotional performances. Voice problems during childhood may reflect on the development of proper capacity to communicate in the adult life.

Children vocal disorders are relatively frequent, affecting 6 to 23% of the children population^3^, ^4^, ^5^. Vocal nodules are the main laryngeal lesions found in children, which pathophysiology is directly associated with vocal abuse^6^^,^^7^. Other predisposing factors include: obstructive nasal manifestations, hearing acuity reduction, velopharyngeal failure, viral or bacterial laryngitis, laryngeal papillomatosis, congenital tumors (hemangiomas, lymphangiomas), minimum structural lesions (asymmetries, cysts, sulci, bridges, microwebs), and others^8^, ^9^.

The range of etiological factors associated with child dysphonia requires early and precise diagnosis, which is not always possible due to numerous factors. One of them is the delay in consulting with a health care professional, and such fact is assigned to the little concern parents have in relation to their children's vocal changes, since children do not have more alarming symptoms involving other systems. Another factor is a delay in the diagnosis and treatment of children dysphonia, associated with little collaboration of the child during the ENT exam, lack of proper instruments to examine the child's larynx, and the difficulties of a detailed exam, even in the hands of experienced professionals. These limitations are due, in part, to anatomical idiosyncrasies of children larynxes, which besides being smaller than that of adults, have the epiglottis more posteriorly positioned, preventing a proper exposure of the vocal folds. Thus, the use of complementary methods in the evaluation of the vocal characteristics of children is of the utmost importance, and it can be carried out through computerized auditory-perception and acoustic analyses. In order for these assessments to be reliable it is necessary to establish previously, well-defined normality standards among the genders and different age ranges. These evaluations complement the ENT exam and can help in the feedback from medical, speech and hearing treatments.

For the subjective voice analysis we used the broadly accepted GRBAS scale, for it is a simple method, geared towards the main vocal characteristics such as the degree of dysphonia (G), roughness (R), breathiness (B), asthenia (A) and speech stress (S)^10^. The subjective assessment in children is important, for some changes considered diseases during voice acoustic analyses in adults are constantly seen in the pediatric population and must be interpreted carefully. According to Martins & Behlau^11^, the sudden vocal attack is an inadequate speech pattern; nonetheless, it is frequently seen in the auditory-perception assessment in children without vocal symptoms, being attributed to the intenseness with which children perform their activities, including vocal emission.

The value of computerized acoustic vocal analysis has been constantly recognized, since, besides providing qualitative data, it also allows for a quantitative analysis of vocal parameters. Computer software, through the use of vocal assessment parameters, are able to provide different vocal assessment parameters, and the most studied ones are the fundamental frequency, shimmer and jitter disturbance measures, and the harmonic/noise ratios. The fundamental frequency (f0 - Hz) corresponds to the number of glottic cycles per second, and 80 to 150 Hz are considered normal for men, 150 to 250 Hz are normal for women and above 250 Hz is normal for children^12^. Jitter represents the variation in frequency periodicity and shimmer represents the periodicity variation in amplitude. Harmonic/noise variation ratios provide an index which associates the harmonic component and the noise component of the acoustic wave.

Voice analysis computer-based software have normative values for these parameters for the adult population considering both genders; however, not for the pediatric population. The need to establish comparative parameters with the normal values of the acoustic analysis in children has been approached by numerous authors. Behlau et al.^13^ assessed 30 children between 8 and 12 years and found a jitter percentage of 2.3% and shimmer of 2.5%. Linders et al.^14^ analyzed vocal samples from 71 children with ages between 7 and 15 years and noticed mean f0 jitter values around 244 Hz and 9.7 for girls and 250 Hz and 10.3 for boys.^15^

The lack of homogeneity in the results from many studies shows the importance of doing additional studies with larger samples. Moreover, it is equally important to include children from lower age ranges in the normative studies since the child's larynx suffer deep and constant changes from birth to adolescence and, consequently, vocal quality changes with growth, being clearer among males. The goal of the present study was to determine the normative vocal parameters in children between 4 and 12 years so that it can be used as a reference by other authors.

## MATERIALS AND METHODS

This study was approved by the Ethics Committee of the institution where it was held (protocol number 2630-07). The vocal samples were obtained from school-aged children in the age range between 4 and 12 incomplete years, from both genders, without vocal symptoms, after consent from the parents or guardians by signing the free and informed consent form. We included 240 children distributed by age in four subgroups as follows: G1 (4 to 5 years, n-60), G2 (6 to 7 years, n-60), G3 (8 to 9 years, n-60) and G4 (10 to 12 years, n-60).

In the study we enrolled only those children without vocal symptoms or other symptoms which could impact the analysis as nasosinusal, respiratory or auditory symptoms. Information on the symptoms were collected by means of a questionnaire given to the parents, who filled out the evaluation protocol with data concerning age; gender; nasosinusal, auditory, vocal and gastroesophageal symptoms; associated respiratory disorders, high vocal demand and exposure to high noise levels.

In order to assess the integrity of the upper airway structures, after filling out the questionnaire, all the children selected were submitted to general otorhinolaryngological exam done by the head researcher, involving the exam of the oral cavity, nasal cavities, rhinopharynx, larynx and ears. For a detailed exam of the vocal folds, the children were submitted to videolaryngoscopy (70°° 8mm rigid scope from Asap, Germany) or by means of the flexible nasal-laryngeal fiberscope (3.3 mm; from Olympus, Japan) in those children who did not tolerate the rigid scope, using the multifunctional video system type XE-30, Eco × -TFT/USB (Germany), recording images on a DVD. Children with vocal fold lesions and those who did not allow endoscopic evaluation were taken off the study.

Continuing with the evaluations, the children were submitted to hearing acuity assessment using transient otoacoustic emissions (ILO – 288, Otodynamic equipment), because it is a fast, painless and important means used for auditory screening. Children without otoacoustic emissions were taken off the study and referred to complementary auditory tests.

The vocal analysis was carried out by the speech and hearing therapists - co-authors of this paper, and included auditory perception evaluations (carried out using the GRBASI scale) and vocal acoustic analysis. For this we used the Multi-Dimensional Voice Program (MDVP) software, model 5105, version 3.1.4, with the Multi-Speech 3700 (Kaypentax, USA), with a Windows-based software, coupled to a microphone with a standard sound board (sound blaster). We used a professional microphone (Shure), placed at a 90° angle in the mouth, keeping a 5cm distance for the utterance of vowel /a/ and at 10 cm for the patient to count from 1 to 10 and for spontaneous speech. The child was instructed to inhale deeply before uttering the sustained vowel /a/, and to do phonation in intensity and loudness levels which would be comfortable for the person doing it, keeping a constant pitch. The tests were done in a silent room, the signal was stored in the computer and pre-processed with the removal of the initial and final unstable portions, standardizing the signals at 3 seconds, the amplitudes between +1 and −1 and the use of an algorithm in order to remove the linear trend, thus preventing recording characteristics to influence acoustic parameters. The acoustic parameters analyzed were: fundamental frequency (f0 - number of cycles/second), jitter percentage (%), shimmer percentage(%), PPQ (Pitch Perturbation Quotient), APQ (Amplitude Perturbation Quotient), SPI (Soft Phonation Index) and NHR (Noise/ Harmony noise). The analyses of these parameters were carried out with the sustained /a/ vowel, eliminating the irregularities in the beginning and end of utterance.

As far as the statistical methodology goes, for the quantitative variables which had a normal distribution we used the variance analyses, followed by the Fukey approach. If not, we used the Kruskall-Wallis. The significance level used was 5%.

## RESULTS

The children were divided in 4 subgroups according to age: 4 to 5 years, 6 to 7 years, 8 to 9 years and 10 to 11 years. In each group we had 30 boys and 30 girls.

The mean values and standard deviations for the vocal parameters extracted from the acoustic analysis are presented on Table 1, according to age and gender. The values for jitter and shimmer percentages, which corresponds respectively to the measures of fundamental frequency perturbation and amplitude perturbation between neighboring cycles, did not show statistically significant differences among the age ranges. Similarly, the values of PPQ (Pitch Perturbation Quotient) and the NHR (Noise Harmonic Ratio) were also kept stable in the different subgroups. The following attributes f0 (fundamental frequency), APQ (Amplitude Perturbation Quotient) and the SPI (Soft Phonation Index) showed a statistically significant difference between the subgroups. With age increase there was a reduction in f0 and in APQ (Amplitude Perturbation Quotient) values. On the other hand, there was an increase in SPI values. In none of these parameters there were gender differences up to the age of 11 years (Table 1).

**Table 1:** Mean and standard deviation of the vocal parameters studied in the different age ranges in both genders.

Age range (years)	4 to 5		6 to 7		8 to 9		10 to 11	
Parameters (Mean and SD)	M F		M F		M F		M F	
Fo	275,09	257,14	243,37	258,93	227,30	230,48	222,49	234,09
*Age p<0.001	4	6	5	2	4	5	8	5
Gender p = 0.32	23,697	29,062	23,022	33,695	21,521	23,799	18,216	15,699
% Jitter	1,710	1,635	1,181	1,725	1,532	1,621	1,709	1,670
Age p=0.60	1,433	1,130	0,798	1,161	1,090	1,075	0,945	0,800
Gender p = 0.34								
% Shimmer	4,366	5,048	4,010	5,152	4,240	4,740	4,011	4,220
Age p=0.19	1,704	1,668	1,425	1,984	1,068	1,959	1,100	1,208
Gender p =0.18								
APQ	3,133	3,543	3,147	3,562	3,255	3,289	2,789	2,858
*Age p=0.06	1,217	1,141	1,609	1,318	1,101	1,251	0,702	0,796
Gender p = 0.13								
PPQ	0,850	0,955	0,722	0,937	0,872	0,959	1,024	1,015
Age p=0.36	0,618	0,646	0,528	0,577	0,563	0,664	0,561	0,486
Gender p= 0.18								
SPI								
*Age p =0.0003	4,998	5,018	5,492	5,476	7,049	6,008	7,911	6,690
	3,905	3,147	2,758	3,639	3,166	2,393	3,185	2,731
Gender p = 0.16								
NHR								
Age p=0.46	0,132	0,135	0,137	0,142	0,141	0,135	0,125	0,134
Gender p = 0.52	0,030	0,022	0,050	0,047	0,051	0,023	0,023	0,031

* Statistically significant parameters (p<0.05).

## DISCUSSION

Vocal assessment in children have particularities which differ from those in adults, such as the little cooperation in tests, the small sizes of laryngeal structures and the vocal characteristics of the similarities between the genders and pre-school aged children, the change in vocal parameters near the time of voice change and the difficulties in defining normative parameters for the vocal samples in the different age ranges. This is extremely important in order to avoid interpretation errors during vocal quality analyses. It is equally important to associate many diagnostic methods in the assessments, since there is not always correlation between the auditory perception evaluation and the nasofibroscopy findings in 33 institutionalized children, in the age range of 6 to 12 years without vocal complaints. Even when including in the sample group only those asymptomatic children, endoscopic tests revealed different functional and organic disorders, which included laryngeal constriction, glottic cleft and vocal nodules. The correlation of auditory perception vocal analysis with endoscopic findings was low. Thus, the authors stress the importance of associating numerous methods in these assessments and in establishing the normative parameters for each one.

Auditory perception vocal analysis require the opinion from a group of experienced professionals, since the definition of whether a voice is normal or altered is not an easy task for experts, let alone the parents of these children, who are responsible for filling out the questionnaires. For this end, the GRBASI scale has been broadly used, nonetheless, the inclusion of computerized acoustic analyses makes this assessment more accurate and less subjective, thus representing an important tool for vocal screening, for it is a simple, fast and reliable method. Nonetheless, the interpretation of samples in this test requires a prior knowledge of the normative standards, not established in the voice analysis software for the pediatric population. In this one, the vocal samples must be colleted with standardization, as well as the proper selection of the sampling group components, and one should exclude those children with comorbidities such as hearing acuity, velopharyngeal failure, nasosinusal obstructive symptoms, repetitious upper airway infections, and others^16^^,^^17^. The sample size is another factor to be considered, having in mind the anatomical individual variations between the different age ranges and genders.

Wertzner et al.^18^ analyzed the vocal characteristics of a group of children with speech disorders, trying to establish a relationship between the articulatory and vocal patterns. For this end, they included 20 children with speech disorder in their series and 20 without it, in the age range between 4 and 10 years. In the results, the mean values of vocal parameters in both groups for the /a/ vowel were, respectively: jitter % between 1.55% and 1.609%; shimmer (dB) between 0.610 and 0.610 and fo between 243 and 246 Hz. A small reduction in the fo values for the /e/ vowel was noticed by the authors in the group of children with speech disorders. The jitter values were very similar to the ones in this study. As we compare these results to the ones found in the present study, we observe that, since the authors did not break the children down according to age, it was not possible to follow the fo drop with the increase in age, and such data is very clear in our results. The f0 drop analysis in the different age ranges indicate the growth in laryngeal structures with age, and such fact was also stressed by Braga et al.^19^, who studied the exclusive behavior of this parameters in the voices of 100 children between 6 and 8 years (50 boys and 50 girls) and found an average f0 value of 249.71 Hz and reduction in this attribute with the increase in age, and there was also a difference between the genders.

Vanzella^20^ assessed vocal samples from 182 children aged between 7 and 10 years. Since it did not include children below 7 years of age, the mean f0 values recorded were around 237.15 Hz, and such values are similar to those seen in this study concerning the corresponding age ranges. The mean value of the jitter % presented by the author was 1.21%, mildly lower than what was found in this study; nonetheless, the shimmer % was mildly higher, in around 8%.

The need to include children younger than 7 years in the series of normative studies is justified by the constant vocal abuse in children recreation by the progressive changes in laryngeal structures in children and the frequent diagnosis of vocal nodules in this population. In this study we have also seen a reduction in the APQ mean values and increase in SPI in the older children, indicating greater vocal emission stability with laryngeal growth. The results from vocal assessments in children aged between 4, 5 and 6 years, held by Capellari & Cielo^21^, used a maximum phonation time (MPT) and acoustic parameters, were also interpreted as inherent to the laryngeal maturation process which occur naturally with the development of the child, and we observed a significant MPT increase and fo reduction in the three age ranges (255.06 Hz, 253.18 Hz and 248 Hz, respectively). The authors also observed that the APQ, PPQ and NHR values were higher at 4 years, and this last parameter was statistically significant. This data was not confirmed in our study and the reduction in the mean APQ values we found proved to be statistically significant only when comparing the children in age ranges near 10 years, keeping stable until 7 years.

Nicollas et al.^22^, trying to establish the acoustic normative parameters in the voices of children, used a relevant series made up 310 children from 6 to12 years, without vocal symptoms or respiratory tract infections. The results presented by the authors were similar to the ones reported in the present study, there was a reduction in fo with age and difference between the genders (boys – 268.9 to 234.42 Hz; girls – 260.92 to 239.43 Hz), without important variations in jitter and shimmer values.

In the population of this study, the acoustic analysis parameters assessed which most established statistical difference were fo, APQ and SPI, and there is a certain homogeneity among the remaining parameters. Changes to vocal qualities become even more evident during adolescence, especially among boys. Between 13 and 15 years of age boys enter voice change, characterized by the fast growth of laryngeal structures, stressing the elongation of vocal folds, a greater angle of the thyroid cartilage sheaths and the differentiation of the vocal ligament structures. These events happen with important changes in voice quality, secondary to the lack of coordination and lack of intrinsic muscle adaptation to the new laryngeal dimensions^23^. These facts justify the need for excluding these children from studies which establish normative vocal parameters.

## CONCLUSIONS

Here, we present the normative values from vocal acoustic parameters of children between 4 and 12 years, and with aging, the most marked changes were f0 and APQ reduction and SPI increase. Changes to the other parameters did not establish statistical difference among the age ranges. We did not find significant differences concerning the vocal parameters studied as far as gender is concerned.
